# Cash Transfers and After-School Programs: A Randomized Controlled Trial for Young Men at Risk of Violence Exposure in Wilmington, Delaware

**DOI:** 10.1007/s11524-024-00838-y

**Published:** 2024-04-18

**Authors:** Christina Plerhoples Stacy, Daniel Teles, Jorge González-Hermoso, Fay Walker, Anna Morgan, Steven Huettner, Rachel L. J. Thornton, Pamela A. Matson

**Affiliations:** 1 Urban Institute, Washington, DC USA; 2 School of Medicine, Johns Hopkins University, Baltimore, MD USA; 3 Nemours Children’s Health, Wilmington, DE USA

**Keywords:** Cash transfers, Youth violence, Urban health

## Abstract

We conducted a randomized controlled trial to determine whether an after-school program paired with a cash transfer (a conditional cash transfer) or a cash transfer alone (an unconditional cash transfer) can help improve health and economic outcomes for young men between the ages of 14 and 17 whose parents have low incomes and who live in neighborhoods with high crime rates. We find that receiving the cash transfer alone was associated with an increase in healthy behaviors (one of our primary outcome composite measures) and that the cash transfer paired with after-school programming was associated with an improvement in the financial health of participants (one of our secondary outcome composite measures). We find no differences in spending on alcohol, marijuana, cigarettes, or other drugs between either the treatment group and the control group. Neither the cash transfer alone nor the programming plus cash transfer had statistically significant effects on our other primary composite measures (physical and mental health or school attendance and disciplinary actions), or our other secondary composite measures (criminal justice engagement or social supports) but in most cases, confidence intervals were too large to rule out meaningful effects. Results suggest that cash transfers hold promise to improve the health of youth without any indication of any adverse effects.

## Introduction

Homicide is the leading cause of death for non-Hispanic Black or African American youth, due in large part to the lasting effects of structural racism, which contribute to factors such as poverty, housing instability, racial wealth gaps, and a lack of prosocial outlets [1–3]. Youth violence is particularly pronounced in Wilmington, Delaware, where more than 3 out of every 1000 teens are injured or killed by gun violence annually—nearly double the rate of cities like Chicago, Illinois, or Trenton, New Jersey, which are known for their high crime rates [4].

In 2014, the Centers for Disease Control and Prevention (CDC) conducted an epidemic investigation in response to the disproportionately high homicide rate in Wilmington, DE compared to every other state [5]. They concluded that most individuals involved in firearm violence were young men with a substantial number of risk factors, including cumulative experience with prior violent injury, unemployment, child welfare investigation, juvenile justice involvement, and adverse school system events (e.g., drop-out, disciplinary actions).

In response, the State of Delaware Department of Health and Social Services (DHSS) began to look at new ways to meet the needs of young men with risk factors identified in the CDC’s report. The state offered opportunities for job training and subsidized employment as well as comprehensive programming that prepared youth for employment. But, there was a low uptake of these opportunities and the administration wanted to try something new. Leadership at DHSS recognized that poor youth engagement in employment opportunities was likely a result of structural inequities that not only impact employment programming participation but also increase vulnerability in the other domains, including school engagement and juvenile justice involvement. Monetary subsidies or cash transfers might help youth overcome barriers to program participation, such as money for transportation, childcare, food, stable housing, appropriate clothing, or meeting other basic needs. Structural issues are both complex and dynamic, with a multi-level, bi-directional influence between youth and their social environment (policies impact families, family functioning impacts youth, etc.), and as a result, the specific barriers faced by any individual youth and family are heterogeneous.

Alternatively, cash transfers alone might help surmount structural barriers. It is possible that youth violence is not caused by youth unemployment; but rather, youth unemployment and violence are correlated and are both caused by larger structural issues. Providing an unconditional cash transfer directly to youth that is not conditional on programming participation may allow them to address their individual and family needs that facilitate thriving in social spaces tailored to their personal goals, including school, and avoid adverse systems interactions, such as justice and/or child welfare investigation involvement. They could also directly improve outcomes by addressing challenges like unstable housing, lack of food security, limited transportation options, and insufficient funds for healthy recreational activities [6–9].

To test these hypotheses, we partnered with Delaware DHSS to implement and study a cash transfer with and without an after-school program. The “Yes! Study” involved a randomized controlled trial where 167 young men between the age of 14 and 17 were assigned to one of three study arms: (1) after-school program paired with a cash transfer that they received if they attended the first few sessions (a partially conditional cash transfer); (2) a cash transfer without any program requirements (an unconditional cash transfer); or (3) a waitlisted group that served as a control group and received no treatment until after the study was complete. Eligibility for the program was based on family income and ZIP code of residence. The after-school program consisted of activities such as tutoring, job training, conflict resolution training, financial coaching, recreational and arts programming, and training for social skills.

We focused on analysis on three primary outcomes: a physical and mental health measure based on survey responses related to mental health and violence victimization, a health behaviors measure based on questions about criminal and other risky behavior, and a measure of school attendance and disciplinary actions based on both survey data and school administrative data. A key theoretical goal of this intervention was a reduction in criminal justice engagement and violence exposure. We created a survey-based composite measure related to criminal justice but consider it a secondary outcome because we did not have the necessary sample size to estimate impacts on low-probability events and we were unable to access reliable administrative data on criminal justice engagement to augment the survey responses.

We find that receiving the UCT was associated with an increase in healthy behaviors, one of our primary outcome composite measures. This means that participants who received the cash transfer were less likely than the control group to do things like drink alcohol, use marijuana, take prescription medication without a prescription, be in a physical fight, carry a weapon, or use a vapor product. Neither the cash transfer alone nor the cash transfer paired with programming had statistically significant effects on our other composite measures for our primary outcomes of interest: physical and mental health or school attendance and disciplinary actions. But, in most cases, confidence intervals were too large to rule out meaningful effects—either positive or negative.

We also find that the cash transfer paired with programming was associated with improved financial health, one of our secondary outcome composite measures. This may be because the after-school programming included financial coaching, which has been shown through experimental data to produce a number of significant effects on a variety of outcomes that contribute to financial health, including financial stress (Theodos, Stacy, and Daniels 2018). There were no statistically significant differences between groups for our other secondary composite measures: criminal justice engagement or social supports.

Quantitative analysis from the survey data showed that participants in the cash transfer-only group spent more money over the past 30 days on electronics, accessories, food, entertainment, and “other items” than the control group. Participants in the cash transfer plus programming group spent less on books and magazines than the control group, but no differently on any other items. There were no statistically significant differences between either treatment group and the control group for spending on alcohol, marijuana, cigarettes, or other drugs, meaning that they did not report purchasing more of these items than the control group.

These results show that cash transfers alone are associated with an increase healthy behaviors and a reduction in risky behaviors for young men at risk of violence exposure, and that cash transfers plus programming are associated with improvements in the financial health of these young men. Given that the sample was small and take-up rates were not 100%, we expect our estimates to be a lower-bound estimate for the effect of the intervention on our outcomes of interest. Our study lacks the power to detect modest but meaningful changes in other composite measures such as overall physical and mental health, school attendance and disciplinary actions, criminal history, and social supports. With more power, these effects may be present. Future research should conduct similar analyses on a larger sample to determine whether these effects are present.

## Methods

We conducted a three-arm RCT and pre-registered our research design with the Open Science Foundation (https://osf.io/wxtsb/). We randomized participants into the following three groups:

1. Cash transfer paired with after-school programming (conditional cash transfer). This group began programming in the fall of 2021 and received six months of after-school curriculum accompanied by $150 a week if they attended enough sessions of the afterschool program to submit all of their relevant documents for the cash transfer (which was generally 2 to 4 sessions). The programming and cash transfer ended in May of 2022. There were 59 programming plus cash transfer participants; 55 excluding dropouts.
2. Cash transfer alone (unconditional cash transfer). This group received $150 weekly for six months beginning in the fall of 2021, ending in May 2022. There were 56 participants in cash transfer only.
3. Control group. This group was waitlisted for programming after the completion of the study. Control group programming began in the summer of 2022 and ran until November 2022. There were 57 control group participants; 56 excluding dropouts.

The after-school programming consisted of tutoring, conflict resolution training, financial coaching, recreational and arts activities, and soft skills training. DHSS provided transportation to and from the program venue, food, and tutoring before all other planned activities; between 3:30 pm and 4:30 pm. Programming took place an office park and retail center in the riverfront area of Wilmington, which was considered by the participants to be a safe, neutral location.

### Study Participants

Young men eligible for the study were between the ages of 14 and 17, lived in families with low-incomes (those eligible for Medicaid), and resided in three Wilmington ZIP codes that DHSS identified as having high rates of violent crime: 19801, 19802, and 19805.

Using Medicaid enrollment data, DHSS identified close to 2000 eligible young men in the spring of 2021 to participate in the study. During summer and fall of 2021, we worked with DHSS to send an introductory flyer, e-mail, and text messages with information about the program and study to all eligible youth.

We implemented different IRB-approved methods to increase enrollment into the study:

- Cold calls to eligible families conducted by the Urban team to explain the study and invite them to enroll.
- A dedicated Facebook page about the study with flyer-style posts and information about how to enroll that was continuously updated.
- A raffle for $250 for enrolled participants who successfully invited other eligible participants to enroll.
- Partnerships with local nonprofit organizations focused on youth development to invite eligible clients to enroll in the study.

However, even with these enhanced recruitment methods, participation did not reach our sample size goal of 225. Due to COVID-19 safety protocols instituted at the time of recruitment, in person recruitment activities were not allowed. These restrictions likely had a significant impact on our final sample size since face-to-face connection is key to gaining trust amongst potential participants. Overall, 172 young men enrolled in the study; 167 excluding dropouts.

### Randomization

Randomization occurred in one batch in October 2021. We used blocked (stratified) randomization to ensure balance across groups in terms of race, ethnicity, and neighborhood. Pairs of siblings were kept in the same treatment or control group. Individuals randomized into the cash transfer plus programming and cash transfer alone had a 2021 start date, and those randomized into the control group were offered to start programming in 2022, after the RCT was completed. All groups, including the waitlisted control group, took all surveys during the same time ranges. Within the first few weeks of enrollment, one youth dropped out of the control group since he was incarcerated and four dropped out of the cash transfer plus programming for unreported reasons.

### Data Collection

We collected data via surveys and through administrative data from the Delaware Department of Health and Social Services on program participation and cash transfer pickup rates, and from the Delaware Department of Education on school attendance and disciplinary actions.

#### Surveys

Participants enrolled in this study were administered a total of six surveys: a baseline survey after enrollment but before programming began (or during the first week of programming), four consecutive monthly surveys, and a final exit survey after the completion of the program and cash transfer.

The baseline and the final exit surveys consisted of a maximum of 107 questions (inclusive of skip patterns). The baseline survey assessed demographics and self-reported school attendance, employment status, saving and spending patterns, financial stress, perceived health status, and criminal justice involvement. The measure of food insecurity was selected from the National Survey of Children’s Health [10]. Five items were used to assess housing instability consistent with the CDC definition including affordability, risk of eviction, and frequent moves [11]. Validated scales were used to assess social support and psychological distress, which was incorporated into the physical and mental health composite [12, 13]. Fifteen items with strong psychometric properties were used to measure violent and non-violent delinquency behaviors [14]. Three items were used to assess self-esteem and two items were to assess future orientation in the domains of fatalism and belief in the future [14, 15]. Self-reported lifetime use and past-30 day frequency of substance use were assessed with measures consistent with those from the Youth Risk Behavior Surveillance System [1].

The monthly survey consisted of 36 questions and was repeated for four consecutive months. Topics included income, employment status, purchasing patterns, family responsibilities and financial contributions to household expenses, food and financial security, delinquency behaviors, criminal justice involvement, substance use, self-esteem, and perceived health status for the previous 30 days. For youth in the cash transfer plus programming group, the monthly survey assessed engagement in programming, including reasons for missing program sessions.

The final exit survey repeated assessments of food insecurity, housing instability, saving and spending patterns, financial stress, social support, psychological distress, self-reported school attendance, employment status, financial stress, perceived health status, substance use, delinquency behaviors, criminal justice involvement, and future orientation. For youth in the cash transfer plus programming group, the exit survey assessed engagement in and perceived usefulness of the program.

Upon survey completion, participants were sent an e-gift card to either their cell phone or email address for time spent participating in the study. Participants received a $20 e-gift card for the baseline survey and each completed monthly survey, and $40 e-gift card for completing the exit survey. In order to increase exit survey response rates, the team increased the value of the gift card ($20 to $40), used a third-party outreach worker to visit participants at their homes to assist with completion of the exit survey, and offered a special event with food at the location of the program sessions where participants were invited to complete the survey. The increased outreach methods led to 126 participants completing the exit survey.

#### Administrative Data

We collected administrative data from the Delaware Department of Health and Social services on program participation and cash transfer pickup, as well as data from the Delaware Department of Education on school attendance and disciplinary actions. We also used Medicaid enrollment data to draw our sample.

### Outcomes of Interest

The primary outcomes of interest for this study include those related to physical and mental health, health behaviors, and school attendance and disciplinary actions (Table 1). The secondary outcomes of interest include criminal history/involvement with the justice system, financial health, and social supports. Despite being a goal of the intervention, criminal history/involvement with the justice system is not a primary outcome in our analysis due to a lack of administrative data and the low frequency of such engagement which makes estimation challenging. Some criminal justice related measures are included in our primary measures of physical and mental health when they relate to injury and health behaviors, such as questions about fighting and carrying weapons.

**Table 1:** Outcomes of interest

Primary outcome composite measures	Survey question or administrative data variable
Physical and mental health	In the past 30 days, how often did you feel nervous?
	In the past 30 days, how often did you feel so nervous that nothing could calm you down?
	In the past 30 days, how often did you feel hopeless?
	In the past 30 days, how often did you feel restless or fidgety?
	In the past 30 days, how often did you feel so restless that you could not sit still?
	In the past 30 days, how often did you feel depressed?
	In the past 30 days, how often did you feel that everything was an effort?
	In the past 30 days, how often did you feel so sad that nothing could cheer you up?
	In the past 30 days, how often did you feel worthless?
	In the past 30 days, how many times have you been seen in an emergency room or ER?
	During the past 30 days, how many times were you in a physical fight in which you were injured and had to be treated by a doctor or nurse?
	During the past 30 days, how many times has someone threatened or injured you with a weapon such as a gun, knife, or club?
	On a scale from “No chance” to “It will happen” what do you think are the chances you will be killed by the age 21?
	I have a lot of good qualities
	I have a lot to be proud of
	I feel loved and wanted
Health behaviors	Have you ever drunk alcohol?
	Have you ever used marijuana?
	During your life, how many times have you taken prescription pain medicine without a doctor’s prescription or differently than how a doctor told you to use it? Count drugs such as codeine, Vicodin, OxyContin, Hydrocodone, and Percocet
	During the past 30 days, how many times were you in a physical fight?
	During the past 30 days, on how many days did you carry a weapon such as a gun, knife or club?
	Have you ever used an electronic vapor product?
School attendance and disciplinary actions	During the past 30 days, how often did you skip school without an officially-excused absence?
	In general, how hard do you try to do your school work well?
	During the past 30 days, how many times were you in a physical fight on school property?
	Have you been high on drugs at school in the past six months?
	Percent of school days attended +
	Any disciplinary incidents + Number of disciplinary incidents + Any severe disciplinary incidents +
	Number of severe disciplinary incidents +
**Secondary outcomes**	
Criminal history/involvement with the justice system	In the past 30 days, did you deliberately damage property that didn’t belong to you?
	In the past 30 days, did you take something from a store without paying for it?
	In the past 30 days, did you use or threaten to use a weapon to get something from someone?
	In the past 30 days, how many times have you been stopped or detained by the police for questioning about your activities? By detained I mean kept waiting or from going on your way by police, but not arrested
Financial health	Do you have a bank account in your name?
	Which of the following statements best describes your household’s ability to afford food you need during the past 30 days?
	During the past 30 days, have you contributed by paying money to another household member, paying certain household bills, or buying things—such as groceries—for the household?
	On a scale of 1 to 7, where 1 is no stress at all and 7 is overwhelming stress, what do you feel is the level of your financial stress today?
	On a scale of 1 to 7 with 1 being never and 7 being all the time, how often does this happen to you—you want to go out to eat, go to a movie or do something else and don’t go because you can’t afford to?
Social supports	There is an adult who is around when I am in need
	I can count on my friends when things go wrong
	My friends really try to help me
	I have friends with whom I can share my joys and sorrows
	I can talk about my problems with my friends
	I have an adult who is a real source of comfort to me
	How much do you feel that adults care about you?
	There is an adult in my life who cares about my feelings
	My family really tries to help me
	I get the emotional help and support I need from my family
	I can talk about my problems with my family
	My family is willing to help me make decisions

All measures are from survey data, except those noted with ^+ ^which are from administrative data from the Delaware Department of Education

To account for multiple outcomes and the probability of a type I error (a “false positive”), we combine individual measures into composite indices, as shown below. This reduces concerns about false positives for individual variables, similar to the methods used by Kling, Liebman, and Katz [16] and Karlan and Valdivia [17] (to see results for each individual measure, see the online appendix).

### Analysis Methods

Our primary method for estimating the impact of the unconditional and conditional cash transfer plus programming on our outcomes of interest is an “intent to treat” model which tests the effect of being offered treatment on outcomes, whether or not the individuals participated in programming or received the cash transfer. This is estimated using the following linear regression model:

$${\text{Y}}_{i }= \alpha + {\beta }_{1}treate{d}_{i}+{\delta X}_{i}+{\epsilon }_{i}$$

where ${Y}_{i}$ is the outcome of interested measured using the exit survey data and administrative data, ${\alpha }_{i}$ is an intercept, $treate{d}_{i}$ is equal to 1 for the group being studied and zero otherwise, ${X}_{i}$ is a set of control variables, and ${\epsilon }_{i}$ is the error term. For control variables, we first include the education level of the participants’ mother, an indicator for whether he was in foster care, his race and ethnicity, ZIP code, and age. We include these prognostic variables as covariates to increase the precision of the effect estimate. We select these control variables based on theory rather than on baseline *t*-tests of differences since choosing covariates based on significance tests for baseline differences can lead to omissions of important covariates and inclusion of irrelevant covariates (de Boer et al. 2015). Second, we include each of our primary and secondary outcomes calculated using the baseline survey and set to zero if the participant did not respond to the baseline survey questions along with dummy variables identifying participants who did not respond to the baseline survey. We initially planned to include baseline survey responses in a fixed-effects model to account for heterogeneity between groups and any issues that arose with balance. However, lower than expected survey response rates for the baseline survey reduced the sample size available for a full fixed-effects model. Including baseline responses where available and dummies where they are not allows us to keep the full sample of outcome survey respondents while accounting for observable differences at baseline.

We use a regression model rather than a *t*-test of sample means both to include control variables and in order to estimate heteroskedastic robust standard errors which account for the likelihood that the variance of the estimated treatment effect is not constant across participants.

We estimate the above model in three ways. First, we estimate the impact with treatment defined as the cash transfer, where only the cash transfer group and the control group are included to estimate an average treatment effect for the cash transfer component alone. Second, we estimate the model with treatment defined as cash transfer plus programming and with the cash transfer only group excluded. Third, we estimate the model with treatment referring to any type of cash transfer (both conditional and unconditional) and equal to one for both the cash transfer only and the cash transfer plus programming group; this allows us to increase the sample size within this intervention component to examine it from a different angle.

We also estimate the “treatment on the treated” or the impact of actually participating in programming. This method allows us to detect effects that may have been drowned out by non-participation in the prior model. However, participants who choose to participate in programming may systematically differ in unobservable ways from those who choose not to participate, which may cause bias in the results. We correct for this potential bias by estimating the complier average causal effect, which uses an instrumental variables approach to correct for this bias [18]. In this approach, randomization into the treatment group is used as an instrument for the actual treatment. For the cash-only group and for analysis of the combined groups, we define the treatment as receiving the cash transfer. For the programming group, we define the treatment as attending at least one-third of sessions, but also ran robustness checks where treatment is defined as the number of sessions attended, attended a single session, attended half the sessions, and attended two third of sessions.

Our initial research plan included the use of fixed-effects models that would incorporate data from both the baseline and exit surveys. Fixed-effects models would have allowed us to remove any time-invariant unobserved heterogeneity that exists for each individual that may be related to their outcomes. This would account for any baseline differences between groups that existed at the beginning of the programming and cash transfer period. Analysis using this method, however, would have relied on a much smaller sample size. While 72% of participants took the exit survey, only 55% took both the baseline and the exit survey. Among the control group, only 42% of participants took both surveys (24 participants). Similarly, we do not use monthly surveys in our data analysis since response rates for the monthly survey were so low and only 21 participants in the control group took both the baseline, outcome, and at least on monthly survey. Using just the outcome survey, we have a 67% response rate for the control group, a 61% response rate for the programming plus cash transfer group and an 89% response rate in the cash transfer only group.

A reverse power analysis revealed that we would not have the power to identify less than 0.51 to 0.29 standard deviations in our key variables. Given that the sample was small and take-up rates were not 100%, we expect our estimates to be attenuated and a lower-bound estimate for the effect of the intervention on our outcomes of interest.

### Group Equivalence at Baseline

To test for potential differences across cohorts even after randomization, we analyzed differences in demographic characteristics and outcome measures at baseline, presented in Table 2. We find that the groups appear balanced across key demographic characteristics included in the administrative data but there were statistically significant differences between groups in many of our key outcome measures. These differences may exist by chance. Or, they may have been produced by differential nonresponse bias across groups. As described above, we include our primary and secondary outcomes calculated using the baseline survey, where available, to account for observable differences between groups.

**Table 2:** Demographic differences at baseline

	All arms	Control	Cash transfer	Cash transfer plus programming
Number of participants after dropout (count)	167	56	56	55
Age at the start of the study (average)	15.8	15.8	15.6 (0.313)	15.9 (0.778)
Black	84%	84%	86% (0.795)	82% (0.770)
White	15%	14%	14% (1.000)	16% (0.764)
Latine	13%	13%	14% (0.784)	13% (0.972)
Living in ZIP code 19,801	16%	20%	16% (0.625)	13% (0.327)
Living in ZIP code 19,802	33%	32%	36% (0.693)	31% (0.890)
Living in ZIP code 19,805	33%	29%	43% (0.117)	40% (0.208)
Number of participants responding to baseline survey (count)	109	28	44	37
Mother is a college graduate	31%	17%	33% (0.199)	40%* (0.078)
Is in foster care	0.08	0.19	0.05* (0.053)	0.03** (0.028)
Physical and mental health	− 0.03	− 0.23	− 0.03 (0.148)	0.10** (0.019)
Health behaviors	− 0.06	0.01	− 0.02 (0.784	− 0.16 0.438)
School attendance and disciplinary actions +	0.21	0.11	0.29** (0.035	0.23 (0.169)
Criminal justice engagement	− 0.03	− 0.17	0.05 (0.128	− 0.03 (0.452)
Financial health	− 0.08	− 0.30	− 0.05** (0.036)	0.07*** (0.003)
Social supports	4.48	4.29	4.65 (0.196)	4.41 (0.720)

Author’s analysis of administrative data from the Delaware Department of Health and Human Services

We ran *t*-tests on the differences between the cash transfer only, cash transfer plus programming, and cash transfer only or programming plus cash transfer groups against the control group, respectively. *p* values for the hypothesis that these differences are different from zero are shown in parentheses. The percent of participants in each zip code does not add up to 100 because some of the participants reported that they did not live in the zip code listed in the administrative data. ^+ ^The school attendance and disciplinary action measure includes data from both the surveys and administrative data from the Delaware Department of Education. We calculate standard errors using heteroskedastic robust standard errors, with p values listed in parentheses. *p < 0.10, **p < 0.05, ***p < 0.01

## Results

### Who Participated in Programming and Who Picked Up the Cash Transfer Card?

Out of the 55 study participants in the cash transfer plus programming group, 39 (71%) attended at least 1 session (Table 3). Of these, 38 (69%) attended enough sessions to receive the cash transfer (this varied by participant and was determined by the number of sessions needed to submit all of their paperwork for the cash transfer), 16 (29%) attended between 1 and 17 sessions (or one-third of all possible sessions), and 23 (42%) attended 18 sessions or more. The average percent of session attended was 35.14%. While these take-up rates may appear low, they are comparable to other after-school programs such as a multisite, after-school program for middle-school students in an urban school district which saw average take-up rates of 38%, and the Urban Alliance Program for which 54% of participants finished their prework and started their internship [19, 20]. Other forms of educational programing, such as financial coaching, have been shown to have take-up rates of 37% and 56%, and participants who took up coaching usually only attended one session [21].

**Table 3:** Treatment take-up

	Cash transfer	Cash transfer plus programming
Participants after dropout	56	55
Received the cash transfer	48 (86%)	38 (69%)
Attended at least 1 session	n/a	39 (71%)
Attended 18 sessions or more (1/3 of sessions)	n/a	23 (42%)
Took the outcome survey	50 (89%)	36 (65%)
Received the cash transfer and took the outcome survey	46 (82%)	32 (58%)
Attended at least 1/3 of programming sessions and took the outcome survey	n/a	21 (38%)

Authors’ analysis of administrative data and survey data

Out of the 111 participants eligible to receive the cash transfer (those in the cash transfer only group plus those in the cash transfer plus programming group), 86 (77%) picked up the card that would enable them to receive the cash transfers. A larger share of the participants in the cash transfer only group picked up the card (86%) than those in the cash transfer plus programming group (69%); and a *t*-test shows this difference is statistically significant at the 0.05% level. This is likely because those in the programming group were only able to pick up the reloadable cash transfer card if they attended at least the first few weeks of programming (enough sessions for them to submit all of their forms for the cash transfer card). Not picking up the card could also reflect challenges communicating with youth after enrolling in the study (which was also present when attempting to get them to take the baseline survey), and in some cases due to lack of trust in systems that may have historically excluded them.

### What Did Participants Spend the Money on?

**Fig. 1:**
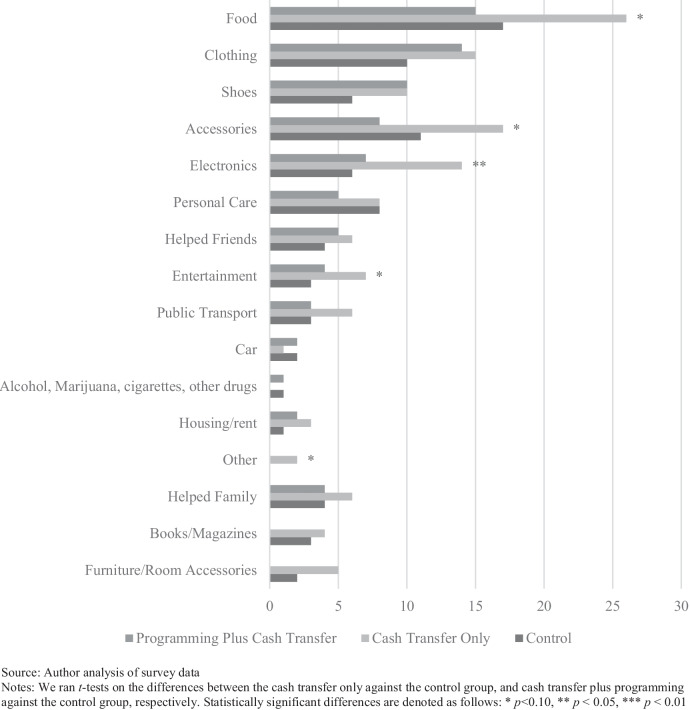
Reported overall spending for treatment groups and control group. Source: Author analysis of survey data

Participants in the cash transfer-only group reported spending more money over the past 30 days on electronics, accessories, food, entertainment, and other items than the control group (Fig. 1). Participants in the cash transfer plus programming group reported spending less on books and magazines than the control group. The programming plus cash transfer group had fewer statistically significant differences from the control group than the cash alone group did. This may be due to lower cash transfer take-up rates of the cash plus programming group and due to the financial coaching components of programming that encouraged the youth to save their money rather than spend it on consumable items. There were no statistically significant differences between either the treatment group and the control group for spending on alcohol, marijuana, cigarettes, or other drugs, meaning that they did not purchase more of these items than the control group did.

### Primary Outcomes

Using both an intent-to-treat and treatment on the treated model, we find that the cash transfer was associated with improved health behaviors for the young men in that group (Table 4). This means that young men who were offered and/or received the cash transfer were less likely to do things like drink alcohol, use marijuana, take prescription medications without a prescription, get in a physical fight, carry a weapon, or use an electronic vapor product. The variables that were statistically significant within this index were prescription medication usage in the ITT model, and marijuana use, prescription medication, and physical fights in the TOT model.

**Table 4:** Impact of conditional and unconditional cash transfers on primary youth outcomes

	Physical and mental health	Health behaviors	School attendance and disciplinary actions^+^
**Intent-to-treat estimates**			
Cash transfer only	0.051 (0.751)	0.334** (0.024)	0.139 (0.410)
Cash transfer plus programming	0.032 (0.872)	0.113 (0.621)	0.070 (0.704)
**Treatment-on-the-treated estimates**			
Treated cash transfer only participants	0.056 (0.708)	0.373** (0.007)	0.177 (0.347)
Treated cash transfer plus programming participants	0.052 (0.846)	0.188 (0.554)	0.189 (0.666)
**Number of observations**			
In cash transfer estimates	88	86	111
In cash plus programming estimates	73	71	107

Authors’ analysis of survey data and education data from the Delaware Department of Education

Treated for the cash-only group is defined as having received the cash transfer. Treated for the cash transfer plus programming group is defined as having attended at least 1/3 of programming sessions and having received the cash transfer. All models include a measure of the individual’s response to the same question on the baseline survey or a dummy variable equal to one if the person did not take the baseline survey along with controls for ZIP code, race and ethnicity, and baseline survey responses to questions about food insecurity, housing stability. ^+ ^The school attendance and disciplinary action measure includes data from both the surveys and administrative data from the Delaware Department of Education. We calculate standard errors using heteroskedastic robust standard errors, with p values listed in parentheses. *p < 0.10, **p < 0.05, ***p < 0.01

Additionally, while the other composite measures were not statistically significant, all of them were in the intended direction (i.e., they indicate a more positive outcome for participants in the cash transfer and cash transfer plus programming group than those in the control group). And while the overall composite measures were not significant for physical or mental health, young men in the cash transfer plus programming group were less likely to visit the ER (in both the ITT and TOT models) and young men in the cash transfer group were less likely to visit the ER and more likely to report that they felt loved and wanted in the TOT model, some of the measures within this composite.

Similarly, while the school attendance and disciplinary actions composite index was not statistically significant for any group or model, participants who received the cash transfer were less likely to get into a fight in school in the TOT model.

### Secondary Outcomes

When looking at our secondary outcomes, we find that participants in the cash transfer plus programming group had higher financial health scores than the control group at outcome in both the intent to treat and the treated on the treated models (Table 5). This result was driven participants’ higher likelihood of having a bank account, lower financial stress, and in the TOT model, a higher likelihood of contributing to household finances. Among the cash transfer-only group, the estimated effect on financial health was not statistically significant at the 0.10 level. For the cash transfer plus programming group, estimates from alternative versions of the TOT model with different thresholds for treatment suggest that impacts on financial health increased with participation in the after-school program, which included a financial education component. We estimated effect sizes that rose from of 0.43 for attending any programming, to 0.62 for attending one-third of the sessions, 0.86 for attending half the sessions, and 1.19 for attending two-thirds of the sessions.

**Table 5:** Intent to treat impact of conditional and unconditional cash transfers on secondary youth outcomes

	Criminal justice engagement	Financial health	Social supports
**Intent-to-treat estimates**			
Cash transfer only	0.079 (0.755)	0.191 (0.195)	0.255 (0.502)
Cash transfer plus programming	0.200 (0.409)	0.377** (0.024)	− 0.221 (0.583)
**Treatment-on-the-treated estimates**			
Treated cash transfer only participants	0.089 (0.713)	0.214 (0.130)	0.285 (0.427)
Treated cash transfer plus programming participants	0.323 (0.301)	0.624** (0.012)	− 0.367 (0.522)
**Number of observations**			
In cash transfer estimates	84	88	85
In cash plus programming estimates	70	74	70

Authors’ analysis of survey data and education data from the Delaware Department of Education

Treated for the cash-only group is defined as having received the cash transfer. Treated for the cash transfer plus programming group is defined as having attended at least 1/3 of programming sessions and having received the cash transfer. All models include a measure of the individual’s response to the same question on the baseline survey or a dummy variable equal to one if the person did not take the baseline survey along with controls for ZIP code, race and ethnicity, and baseline survey responses to questions about food insecurity, housing stability. We calculate standard errors using heteroskedastic robust standard errors, with p values listed in parentheses *p < 0.10, **p < 0.05, ***p < 0.01

Although the other composite measures were not significant, individual measures within these composites were. For instance, young men in the cash transfer-only group were more likely to report that they had a friend they can share their joys and sorrows with (in the TOT model), and participants in the cash transfer plus programming group were more likely to say that they could talk about their problems with their family.

## Discussion

Cash transfers designed to improve outcomes for people in communities with high rates of poverty and violence often prove to be difficult to implement from a political perspective. Some opponents believe that people who need financial assistance are untrustworthy stating that their financial position reflects a moral failing rather than a societal one [22]. Therefore, it is difficult for policymakers to garner support for such policies. Rigorous evidence can provide one avenue to support cash transfer policies.

This study shows that providing cash transfers directly to youth who have a high risk of violence exposure encourages healthier behaviors. Specifically, we find that offering an unconditional cash transfer of $150 each week was associated with healthier behaviors among participants (such as reducing drug and alcohol use and physical fights), and that participants that were offered a cash transfer plus after-school programming showed improvements in financial health.

This transfer was novel in that it was provided directly to youth, and that it was relatively large; each young man in the study received $3600 over 6 months (or $600 per month), which relative to the control group’s average income of $240 per month, represented a large sum. The cash transfer also represented a large sum compared to participants’ family income, which for Medicaid eligible families, is an average of $4611/month [23]. That being said, the total amount may not be a large amount given the overall costs of goods, housing, transportation, and services.

These results add to the body of evidence showing positive impacts from cash transfers. Prior research shows that unconditional cast transfers reduce hospitalizations and criminalized activity and improve nutrition, mental health, school attendance and grades, psychological well-being, and the probability of healthy birth weights [24–28]. More recently, early studies of guaranteed income cash transfers in the USA have shown that cash transfer recipients experienced lower rates of income volatility, food insecurity, and improved mental health, energy, and physical functioning [9, 29].

Our research builds more directly on research focused on impacts for young people. For instance, the PROGRESA/Oportunidades conditional cash transfer in Mexico had positive long-term effects on schooling for children in the families that received the transfer [6, 30]. Cash transfers have also been found to reduce school dropout rates [31], improve youth mental health [32, 33], reduce illness rates [34], reduce the likelihood of anemia, help youth grow more quickly [34], reduce child poverty [35], lengthen life spans [36], increase schooling [36], reduce the likelihood of being underweight [36], increase the likelihood of enrolling in school [37], reduce child labor [37, 38], increase psychosocial well-being [39], reduced exposure to violence [40], and increase incomes in adulthood [36]. Studies have shown that these effects are strongest for youth in families with the lowest incomes [37].

The spending results align with studies showing that cash transfer recipients are not more likely to consume temptation goods, such as drugs and alcohol [9, 26, 31, 41–44]. A meta-analysis of studies from Latin America, Africa, and Asia showed that on average cash transfers have a significant *negative* effect on total expenditures on temptation goods, equal to − 0.18 standard deviations [44]. Our research expands these findings to the USA and to young people.

Our study is one of only a few that have compared conditional and unconditional cash transfers in the same study. Prior research has not come to a clear consensus on which type works better. Baird, McIntosh, and Ozler [31] compared a conditional and unconditional cash transfer in Malawi targeted at adolescent girls. They found that the conditional cash transfer was more effective at reducing school dropout and improving English reading comprehension. However, the unconditional cash transfer proved more successful at improving non-school outcomes such as reducing pregnancy and early marriage. In rural Burkina Faso, Akresh, de Walque, and Kazianga [30] found that for school enrollment and most child health outcomes, conditional cash transfers outperformed unconditional cash transfers. Premand and Barry [45] studied a program in Niger and found that cash transfers alone did not alter parenting practices and did not improve early childhood development outcomes but did improve dietary diversity at the household level (but not for children). Finally, McIntosh and Zeitlin [46] studied a workforce training program in Rwanda and found that, while the workforce training program was successful in improving a number of core outcomes, cost-equivalent cash transfers improved the same outcomes as well as consumption, income, and wealth. We find greater improvement in health behavior from the cash transfer alone, but greater improvement in financial health for young men in the program.

Impacts on health behavior may have been larger for the cash-only group because participation in that group was greater—86% for the cash-only group and only 69 percent for the cash plus programming group. And we suspect that the young men who were reached by the unconditional cash transfer but not the conditional one had fewer supports at home since many program attendees reported that they only showed up because their parents made them do so. Other studies have found that youth who drop out of after-school programming are more at risk in terms of drug use and truancy than those who do not drop out, suggesting that these programs miss the young people who need supports the most [47]. Unconditional cash transfers may be better at reaching these young people.

Impacts on financial health may have been stronger for programming plus cash transfer participants because of the financial education component of the programming. Participants reported that the financial education helped them to better save their money, and findings from the TOT analysis with different dosage sizes confirmed that the more sessions a participant attended, the greater positive impact it had on their financial health. And, the financial coaching model used by the organization that provided this component of the intervention, $tand By Me, has been shown to have positive effects on financial well-being, both in their perceptions of progress and on various credit metrics [48]. But financial education was only a component of programming, so regular attendance at programming or the cash transfer may have reinforced or worked in concert with the financial component to yield these results. Unfortunately, the small sample size for our study prevented us from identifying smaller or more heterogeneous impacts. We failed to find statistically significant impacts (at even the 10% level) on our primary outcome composite measures for physical and mental health and school attendance and disciplinary actions. We also failed to find statistically significant impacts on two of our secondary outcome composite measures: criminal justice engagement and social supports. This may be due to the small sample size and less than full participation and survey response rates. Low enrollment rates in the study might imply challenges to scaling it. However, they might also reflect distrust of researchers, in which case a cash transfer and/or after-school program that is not part of a study might be more effective at recruiting participants. Or, low enrollment could reflect distrust of the State. In that case, any programs run by the state may have just as much of a challenge with participation. Or, low enrolment might have been driven by COVID since it is harder to recruit people into programs when it cannot be done face to face. In that case, future enrollment whether as part of a study or not is likely to be much more successful since in-person recruitment is now possible.

While the results are limited due to power, they show that such initiatives hold promise to improve the lives of youth. And, none of our findings suggest that youth used their cash transfer for nefarious purchases or that they increased in risky behaviors, at least allaying concerns about negative impacts. Future research should expand the sample size on such an intervention to determine whether the effects we were starting to see here are statistically significant with more power.

## Appendix Tables 6, 7, 8, 9, 10 and 11

**Table 6:** Estimated impact on components of physical and mental health

	Cash only			Programming plus cash			Any cash		
	ITT estimate (*p*-value)	TOT estimate (*p*-value)	Obs	ITT estimate (*p*-value)	TOT estimate (*p*-value)	Obs	ITT estimate (*p*-value)	TOT estimate (*p*-value)	Obs
In the past 30 days, how often did you feel nervous?^i^	0.335 (0.171)	0.384 (0.112)	81	0.465 (0.247)	0.756 (0.176)	67	0.344 (0.143)	0.394 (0.100)	114
In the past 30 days, how often did you feel so nervous that nothing could calm you down?^i^	0.062 (0.774)	0.071 (0.735)	81	− 0.094 (0.764)	− 0.140 (0.717)	64	0.063 (0.752)	0.073 (0.724)	112
In the past 30 days, how often did you feel hopeless?^i^	0.061 (0.788)	0.07 (0.751)	81	0.000 (0.999)	0.000 (0.999)	66	-0.014 (0.947)	-0.016 (0.941)	114
In the past 30 days, how often did you feel restless or fidgety?^i^	0.133 (0.676)	0.153 (0.624)	81	− 0.094 (0.815)	− 0.154 (0.776)	66	0.029 (0.920)	0.034 (0.911)	114
In the past 30 days, how often did you feel so restless that you could not sit still?^i^	0.076 (0.744)	0.087 (0.702)	80	− 0.01 (0.980)	− 0.016 (0.975)	66	0.083 (0.705)	0.095 (0.673)	113
In the past 30 days, how often did you feel depressed?^i^	0.076 (0.740)	0.087 (0.696)	81	− 0.211 (0.438)	− 0.316 (0.343)	66	0.016 (0.937)	0.018 (0.930)	113
In the past 30 days, how often did you feel that everything was an effort?^i^	0.066	0.076	79	− 0.293	− 0.427	63	− 0.133	− 0.152	110
	(0.870)	(0.847)		(0.532)	(0.454)		(0.695)	(0.660)	
In the past 30 days, how often did you feel so sad that nothing could cheer you up?^i^	− 0.108 (0.590)	− 0.123 (0.520)	80	− 0.032 (0.917)	− 0.046 (0.898)	63	− 0.127 (0.481)	− 0.145 (0.428)	111
In the past 30 days, how often did you feel worthless?^i^	− 0.09 (0.733)	− 0.099 (0.688)	80	− 0.192 (0.518)	− 0.285 (0.431)	65	− 0.137 (0.539)	− 0.153 (0.492)	112
In the past 30 days, how many times have you been seen in an emergency room or ER?^i^	− 0.203 (0.105)	− 0.227* (0.051)	85	− 0.250* (0.085)	− 0.413** (0.044)	68	− 0.178* (0.083)	− 0.202* (0.050)	116
During the past 30 days, how many times were you in a physical fight in which you were injured and had to be treated by a doctor or nurse?^i^	− 0.089 (0.350)	− 0.101 (0.270)	79	0.195 (0.592)	0.316 (0.511)	65	0.036 (0.832)	0.041 (0.812)	111
During the past 30 days, how many times has someone threatened or injured you with a weapon such as a gun, knife, or club?^i^	− 0.057 (0.738)	− 0.064 (0.694)	83	0.101 (0.597)	0.163 (0.539)	68	0.003 (0.980)	0.004 (0.978)	117
On a scale from “No chance” to “It will happen” what do you think are the chances you will be killed by the age 21?^i^	0.076 (0.664)	0.086 (0.610)	79	0.091 (0.746)	0.143 (0.687)	65	0.142 (0.400)	0.164 (0.346)	110
I have a lot of good qualities	0.193 (0.493)	0.218 (0.412)	81	0.311 (0.287)	0.502 (0.185)	68	0.266 (0.232)	0.303 (0.179)	115
I have a lot to be proud of	0.241 (0.435)	0.274 (0.358)	81	0.256 (0.449)	0.413 (0.362)	68	0.254 (0.341)	0.289 (0.290)	115
I feel loved and wanted	0.387 (0.149)	0.438* (0.081)	81	0.386 (0.255)	0.580 (0.154)	67	0.417* (0.079)	0.477* (0.048)	114
Physical and mental health composite	0.051 (0.751)	0.056 (0.708)	88	0.032 (0.872)	0.052 (0.846)	73	0.054 (0.714)	0.061 (0.682)	123

Authors’ analysis of survey data

All models include a measure of the individual’s response to the same question on the baseline survey or a dummy variable equal to 1 if the person did not take the baseline survey, along with controls for ZIP code, race and ethnicity, and baseline survey responses to questions about food insecurity and housing stability. We calculate standard errors using heteroskedastic robust standard errors, with *p* values listed in parentheses

^i^In the composite measure, we use an inverted value (maximum value minus response) so that a positive change corresponds with an improvement in physical and mental health

**p* < 0.10, ***p* < 0.05, ****p* < 0.01

**Table 7:** Estimated impact on components of health behaviors

	Cash only			Programming plus cash			Any cash		
	ITT estimate (*p*-value)	TOT estimate (*p*-value)	Obs	ITT estimate (*p*-value)	TOT estimate (*p*-value)	Obs	ITT estimate (*p*-value)	TOT Estimate (*p*-value)	Obs
During the past 30 days, on how many days did you have at least one drink of alcohol?^i^	Not estimated^ii^								
During the past 30 days, on how many days did you use marijuana?^i^	− 0.311 (0.122)	− 0.348* (0.070)	85	− 0.389 (0.124)	− 0.586* (0.071)	69	− 0.268 (0.153)	− 0.305 (0.113)	118
During your life, how many times have you taken prescription pain medicine without a doctor’s prescription or differently than how a doctor told you to use it?^i^	− 0.312** (0.016)	− 0.352*** (0.003)	83	− 0.038 (0.863)	− 0.061 (0.837)	69	− 0.172 (0.191)	− 0.195 (0.147)	117
During the past 30 days, how many times were you in a physical fight?^i^	− 0.420 (0.102)	− 0.476* (0.053)	83	− 0.051 (0.888)	− 0.077 (0.866)	67	− 0.218 (0.343)	− 0.250 (0.293)	116
During the past 30 days, on how many days did you carry a weapon such as a gun, knife or club?^i^	− 0.098 (0.229)	− 0.110 (0.154)	80	0.046 (0.756)	0.071 (0.711)	66	− 0.027 (0.733)	− 0.031 (0.706)	114
Have you ever used an electronic vapor product?^i^	− 0.017 (0.899)	− 0.019 (0.881)	85	− 0.074 (0.554)	− 0.114 (0.478)	70	− 0.025 (0.818)	− 0.028 (0.797)	119
Health behaviors composite	0.334** (0.024)	0.373** (0.007)	86	0.113 (0.621)	0.188 (0.554)	71	0.201(0.163)	0.227 (0.122)	121

Authors’ analysis of survey data

All models include a measure of the individual’s response to the same question on the baseline survey or a dummy variable equal to 1 if the person did not take the baseline survey, along with controls for ZIP code, race and ethnicity, and baseline survey responses to questions about food insecurity and housing stability. We calculate standard errors using heteroskedastic robust standard errors, with *p* values listed in parentheses

^i^In the composite measure, we use an inverted value (maximum value minus response) so that a positive change corresponds with an improvement in health behaviors

^ii^Not estimated because only one respondent reported having a drink of alcohol in the last 30 days

**p* < 0.10, ***p* < 0.05, ****p* < 0.01

**Table 8:** Estimated impact on components of school attendance and disciplinary actions

	Cash only			Programming plus cash			Any cash		
	ITT estimate (*p*-value)	TOT estimate (*p*-value)	Obs	ITT estimate (*p*-value)	TOT estimate (*p*-value)	Obs	ITT estimate (*p*-value)	TOT Estimate (*p*-value)	Obs
During the past 30 days, how often did you skip school without an officially-excused absence?^i^	− 0.073 (0.833)	− 0.083 (0.803)	81	− 0.176 (0.646)	− 0.282 (0.580)	66	− 0.042 (0.888)	− 0.048 (0.875)	113
In general, how hard do you try to do your school work well?	− 0.001 (0.995)	− 0.002 (0.994)	82	− 0.041 (0.874)	− 0.066 (0.850)	67	− 0.054 (0.759)	− 0.062 (0.733)	115
During the past 30 days, how many times were you in a physical fight on school property?^i^	− 0.433 (0.129)	− 0.491* (0.066)	81	− 0.128 (0.720)	− 0.204 (0.668)	67	− 0.264 (0.299)	− 0.301 (0.247)	114
Have you been high on drugs at school in the past six months?^i^	0.004 (0.964)	0.004 (0.957)	86	− 0.050 (0.293)	− 0.081 (0.209)	71	− 0.007 (0.906)	− 0.008 (0.895)	120
Percent of school days attended	− 3.250 (0.427)	− 4.220 (0.366)	103	− 2.770 (0.516)	− 8.040 (0.473)	100	− 3.68 (0.282)	− 5.55 (0.243)	153
Any disciplinary incidents^i^	− 0.085 (0.430)	− 0.111 (0.369)	103	− 0.081 (0.491)	− 0.235 (0.433)	100	− 0.084 (0.374)	− 0.127 (0.856)	153
Any severe disciplinary actions^i^	− 0.048 (0.644)	− 0.062 (0.599)	103	− 0.020 (0.857)	− 0.058 (0.836)	100	− 0.028 (0.761)	− 0.042 (0.740)	153
School attendance and disciplinary actions composite	0.139 (0.410)	0.177 (0.347)	111	0.070 (0.704)	0.189 (0.666)	107	0.072 (0.615)	0.106 (0.584)	163

Authors’ analysis of survey data and education data from the Delaware Department of Education

All models include a measure of the individual’s response to the same question on the baseline survey or a dummy variable equal to 1 if the person did not take the baseline survey, along with controls for ZIP code, race and ethnicity, and baseline survey responses to questions about food insecurity and housing stability. We calculate standard errors using heteroskedastic robust standard errors, with *p* values listed in parentheses

^i^In the composite measure, we use an inverted value (maximum value minus response) so that a positive change corresponds with an improvement in school attendance and disciplinary actions

**p* < 0.10, ***p* < 0.05, ****p* < 0.01

**Table 9:** Estimated impact on components of criminal justice engagement: intent-to-treat estimates

	Cash only					Programming plus cash			Any cash		
	ITT estimate (*p*-value)		TOT estimate (*p*-value)		Obs	ITT estimate (*p*-value)	TOT estimate (*p*-value)	Obs	ITT estimate (*p*-value)	TOT estimate (*p*-value)	Obs
In the past 30 days, did you deliberately damage property that didn’t belong to you?^i^	− 0.073 (0.263)		− 0.083 (0.189)		82	− 0.058 (0.413)	− 0.087 (0.311)	68	− 0.056 (0.269)	− 0.064 (0.221)	115
In the past 30 days, did you take something from a store without paying for it?^i^	0.018 (0.749)		0.020 (0.711)		83	− 0.021 (0.452)	− 0.034 (0.369)	69	0.001 (0.970)	0.002 (0.967)	117
In the past 30 days, how many times have you been stopped or detained by the police for questioning about your activities?^i^	− 0.003 (0.982)		− 0.004 (0.979)		81	− 0.081 (0.651)	− 0.129 (0.577)	67	− 0.043 (0.688)	− 0.049 (0.654)	114
In the past 30 days, did you use or threaten to use a weapon to get something from someone?^i^	Not estimated^+^										
Criminal justice engagement composite	0.079 (0.755)		0.089 (0.713)		84	0.200 (0.409)	0.323 (0.301)	70	0.114 (0.522)	0.130 (0.473)	118

Author’s analysis of survey data

All models include a measure of the individual’s response to the same question on the baseline survey or a dummy variable equal to 1 if the person did not take the baseline survey, along with controls for ZIP code, race and ethnicity, and baseline survey responses to questions about food insecurity and housing stability. We calculate standard errors using heteroskedastic robust standard errors, with *p* values listed in parentheses

^ii^Not estimated because only no respondents reported using or threatening to use a weapon in the last 30 days

^i^In the composite measure, we use an inverted value (maximum value minus response) so that a positive change corresponds with an improvement in criminal justice engagement

**p* < 0.10, ***p* < 0.05, ****p* < 0.01

**Table 10:** Estimated impact on components of financial health: intent-to-treat estimates

	Cash only			Programming plus cash			Any cash		
	ITT estimate (*p*-value)	TOT estimate (*p*-value)	Obs	ITT estimate (*p*-value)	TOT estimate (*p*-value)	Obs	ITT estimate (*p*-value)	TOT Estimate (*p*-value)	Obs
Do you have a bank account in your name?	0.088 (0.530)	0.100 (0.457)	83	0.350** (0.027)	0.551** (0.008)	68	0.211* (0.087)	0.242* (0.055)	115
Which of the following statements best describes your household’s ability to afford food you need during the past 30 days?	− 0.017 (0.909)	− 0.019 (0.894)	86	− 0.060 (0.744)	− 0.097 (0.690)	69	0.028 (0.835)	0.032 (0.816)	119
During the past 30 days, have you contributed by paying money to another household member, paying certain household bills, or buying things—such as groceries—for the household?	0.196 (0.134)	0.219* (0.084)	83	0.222 (0.099)	0.355* (0.058)	68	0.218** (0.043)	0.249** (0.024)	116
On a scale of 1 to 7, where 1 is no stress at all and 7 is overwhelming stress, what do you feel is the level of your financial stress today? ^i^	− 3.700 (0.626)	− 4.350 (0.553)	72	− 20.240** (0.006)	− 34.190*** (0.002)	62	− 10.460 (0.130)	− 12.240* (0.081)	102
On a scale of 1 to 7 with 1 being never and 7 being all the time, how often does this happen to you: you want to go out to eat, go to a movie or do something else and don’t go because you can’t afford to?^i^	− 8.82 (0.242)	− 9.88 (0.164)	74	− 13.62 (0.190)	− 20.66 (0.103)	62	− 8.95 (0.219)	− 9.66 (0.166)	103
Financial health composite	0.191 (0.195)	0.214 (0.130)	88	0.377** (0.024)	0.624** (0.012)	74	0.288** (0.025)	0.324** (0.012)	124

Authors’ analysis of survey data

All models include a measure of the individual’s response to the same question on the baseline survey or a dummy variable equal to 1 if the person did not take the baseline survey, along with controls for ZIP code, race and ethnicity, and baseline survey responses to questions about food insecurity and housing stability. We calculate standard errors using heteroskedastic robust standard errors, with *p* values listed in parentheses

^i^Responses coded out of 100. In the composite measure, we use an inverted value (maximum value minus response) so that a positive change corresponds with an improvement in financial health

**p* < 0.10, ***p* < 0.05, ****p* < 0.01

**Table 11:** Estimated impact on components of social supports: intent-to-treat estimates

	Cash only			Programming plus cash			Any cash		
	ITT estimate (*p*-value)	TOT estimate (*p*-value)	Obs	ITT estimate (*p*-value)	TOT estimate (*p*-value)	Obs	ITT estimate (*p*-value)	TOT estimate (*p*-value)	Obs
There is an adult who is around when I am in need	0.260 (0.681)	0.291 (0.628)	84	0.316 (0.647)	0.515 (0.585)	68	0.413 (0.441)	0.466 (0.390)	118
I can count on my friends when things go wrong	0.153 (0.734)	0.172 (0.689)	85	− 0.036 (0.951)	− 0.058 (0.941)	69	0.099 (0.795)	0.112 (0.772)	119
My friends really try to help me	0.254 (0.550)	0.284 (0.479)	84	− 0.035 (0.931)	− 0.053 (0.916)	68	0.131 (0.708)	0.149 (0.675)	117
I have friends with whom I can share my joys and sorrows	0.709 (0.099)	0.793* (0.050)	84	− 0.305 (0.563)	− 0.456 (0.481)	67	0.346 (0.362)	0.393 (0.312)	117
I can talk about my problems with my friends	0.059 (0.882)	0.066 (0.863)	84	− 0.355 (0.436)	− 0.532 (0.354)	67	− 0.062 (0.862)	− 0.070 (0.846)	117
I have an adult who is a real source of comfort to me	0.121 (0.787)	0.136 (0.753)	84	0.002 (0.997)	0.003 (0.996)	67	0.208 (0.588)	0.236 (0.548)	117
There is an adult in my life who cares about my feelings	0.391 (0.387)	0.437 (0.312)	84	− 0.534 (0.388)	− 0.872 (0.334)	68	0.114 (0.773)	0.129 (0.749)	118
My family really tries to help me	0.064 (0.887)	0.071 (0.868)	84	− 0.246 (0.673)	− 0.402 (0.619)	68	0.193 (0.628)	0.218 (0.590)	118
I get the emotional help and support I need from my family	0.186 (0.693)	0.208 (0.643)	84	− 0.026 (0.960)	− 0.038 (0.952)	67	0.246 (0.521)	0.279 (0.476)	117
I can talk about my problems with my family	0.481 (0.331)	0.538 (0.247)	85	0.699 (0.162)	1.145* (0.086)	67	0.585 (0.153)	0.667 (0.109)	117
My family is willing to help me make decisions	0.177 (0.694)	0.198 (0.645)	84	− 0.094 (0.812)	− 0.140 (0.777)	67	0.173 (0.627)	0.196 (0.588)	117
Social support scale	0.255 (0.502)	0.285 (0.427)	85	− 0.221 (0.583)	− 0.367 (0.522)	70	0.180 (0.570)	0.203 (0.527)	120

Authors’ analysis of survey data

All models include a measure of the individual’s response to the same question on the baseline survey or a dummy variable equal to 1 if the person did not take the baseline survey, along with controls for ZIP code, race and ethnicity, and baseline survey responses to questions about food insecurity and housing stability. We calculate standard errors using heteroskedastic robust standard errors, with *p* values listed in parentheses

**p* < 0.10, ***p* < 0.05, ****p* < 0.01
